# A Review on Headaches Due to COVID-19 Infection

**DOI:** 10.3389/fneur.2022.942956

**Published:** 2022-07-12

**Authors:** Mansoureh Togha, Seyedeh Melika Hashemi, Nooshin Yamani, Fahimeh Martami, Zhale Salami

**Affiliations:** ^1^Headache Department, Iranian Center of Neurological Researches, Institute of Neuroscience, Tehran University of Medical Sciences, Tehran, Iran; ^2^Headache Department, Neurology Ward, Sina Hospital, School of Medicine, Tehran University of Medical Sciences, Tehran, Iran; ^3^Neurology Department, Zanjan University of Medical Sciences, Zanjan, Iran

**Keywords:** COVID-19, headache, SARS-CoV-2, headache disorders, neuro-inflammation

## Abstract

Since December 2019, the time when the severe acute respiratory syndrome coronavirus 2 (SARS-CoV-2) was spotted, numerous review studies have been published on COVID-19 and its neuro invasion. A growing number of studies have reported headaches as a common neurological manifestation of COVID-19. Although several hypotheses have been proposed regarding the association between headache and the coronavirus, no solid evidence has been presented for the mechanism and features of headache in COVID-19. Headache also is a common complaint with the omicron variant of the virus. COVID-19 vaccination also is a cause of new-onset headaches or aggravation of the previous headache in migraine or tension headache sufferers. In this review study, the types of headaches reported in previous studies and their possible pathogenic mechanisms are outlined. To accomplish this objective, various types of headaches are classified and their patterns are discussed according to ICHD-3 diagnostic criteria, including, headaches attributed to systemic viral infection, viral meningitis or encephalitis, non-infectious inflammatory intracranial disease, hypoxia and/or hypercapnia, cranial or cervical vascular disorder, increased cerebrospinal fluid (CSF) pressure, refractive error, external-compression headache, and cough headache. Then, their pathogeneses are categorized into three main categories, direct trigeminal involvement, vascular invasion, and inflammatory mediators. Furthermore, persistent headache after recovery and the predictors of intensity is further investigated. Post-vaccination headache is also discussed in this review.

## Introduction

In December 2019, a local outbreak of acute respiratory infection known as “severe acute respiratory syndrome coronavirus 2 (SARS-CoV-2)” was first reported from Wuhan, China, which soon caused the current pandemic and therefore drew global attention due to its enormous potential for infectivity. In February 2020, the World Health Organization (WHO) named the disease coronavirus disease 2019 (COVID-19).

The manifestations of this virus extend far beyond the respiratory tract including neurological complications (1). Neurological complications reported being associated with COVID-19 range from mild headache and dizziness to severe conditions such as stroke and seizure (2, 3). The results of a systematic review and meta-analysis indicated that the cumulative prevalence of headache in COVID-19 was 25.2% (4). There is some evidence highlighting that headache is the most common neurological manifestation, which can occasionally be seen alone as the first sign of the disease (5–8). Apart from the headaches associated with covid-19 infection, an increased number of headaches among the general population and those with a previous history of headaches could be referred to the lifestyle changes in the covid-19 era (9–12). The other cause of headache at present is vaccination against covid-19 (13–15). According to the results of a recent meta-analysis, the overall prevalence of headache after vaccination with Pfizer-BioNTech vaccine was about 35% (6).

As the most common non-respiratory symptom, understanding the different types and underlying mechanisms of headache associated with COVID-19 may have clinical implications. To the best of our knowledge, a limited number of reviews have been published addressing headaches from different aspects of COVID-19. Therefore, this review aimed to represent the most common headache types related to the COVID-19 infection and discuss their possible underlying pathophysiology and treatment.

## Headaches Secondary to SARS-CoV-2 Infection

The most common headache types related to the current pandemic, according to their ICHD-3 classification codes, are as follows.

### Acute Headache Attributed to Systemic Viral Infection (9.2.2.1)

To fulfill the diagnostic criteria of acute headache attributed to systemic infection according to the ICHD-3, an individual must have headache attacks of diffuse and/or moderate to severe intensity that has developed, worsened, and improved in temporal relation and parallel with the diagnosed systemic viral infection in the absence of meningitis or encephalitis (16).

Headache occurs commonly in COVID-19 patients and headaches related to COVID-19 infection may have features of either migraine or tension-type headache (TTH) or both. Headache features are not limited to only one phenotype (17–20).

In the omicron variant of the virus, headache complaint is very common and accounted for the second most probable symptom after upper respiratory problems (21).

### Headache Attributed to Viral Meningitis or Encephalitis (9.1.2)

Headache attributed to viral meningitis or encephalitis is described as holocranial or nuchal headache typically with fever and neck stiffness that is variably associated, according to the extent of the infection, with neurologic symptoms in patients with a confirmed diagnosis of meningitis or encephalitis (16).

Headache attributed to viral encephalitis is diagnosed in the presence of multifocal or generalized brain edema and/or at least one of the following: focal neurologic deficit, seizure, and altered mental state (16). While enterovirus, herpes simplex virus (HSV), varicella-zoster virus (VZV), cytomegalovirus (CMV), and influenza virus are the most common causes of acute inflammation of the brain and encephalitis, many other viruses affecting the respiratory tract such as severe acute respiratory syndrome coronavirus (SARS-CoV) and the Middle East respiratory virus (MERS-CoV) can cause brain edema and inflammation (22, 23). Headaches are usually diffuse, severe, throbbing, or pressing with the focus in the frontal or retro-orbital region (16). Viral encephalitis may also cause mild signs and symptoms such as fever and mild headache or may be asymptomatic (24).

Meningeal inflammation is suspected in cases suffering from holocranial or hemicranial headache with fever and neck stiffness that exacerbates with activity and Valsalva maneuver and is accompanied by photophobia, nausea, or vomiting less commonly (25). The diagnosis must be confirmed by neuroimaging evaluation, which exclusively shows enhancement of leptomeninges (16). Moriguchi et al. (26) reported the first case of meningitis/encephalitis associated with SARS-CoV-2 in Japan in late February 2020, a 24-year-old man with chief complaints of headache, generalized fatigue, and fever in whom SARS-CoV-2 RNA was detected in the cerebrospinal fluid by genome sequencing. As the COVID-19 pandemic progressed, reports of encephalitis associated with COVID-19 were increased (27–29). The results of a systematic review on the neurological manifestation of COVID-19 indicated that acute viral meningitis/encephalitis was the main presumed diagnosis among the sample of 409 patients diagnosed with COVID-19 infection (30).

### Headache Attributed to Other Non-infectious Inflammatory Intracranial Diseases (7.3.3)

Cytokine release is involved in headache pathogenesis during COVID-19 infection and plays a role in the modulation of the pain threshold. Headache provoked by cytokine storm has been described to occur between the 7th and 10th day of the disease onset and could be diagnosed as headache attributed to other non-infectious inflammatory intracranial disease (7.3.3) according to ICHD-3 criteria (16, 31).

To explore the role of systemic circulating inflammatory molecules and their clinical relations in COVID-19 patients, Bolay et al. (32) conducted a cross-sectional study on 88 hospitalized COVID-19 patients. They found that the serum level of high mobility group box-1 (HMGB1), Nod-like receptor pyrin domain-containing 3 (NLRP3), angiotensin-converting enzyme 2 (ACE2), and IL-6 was significantly higher among COVID-patients with headache compared patients without it. They concluded that the increased levels of mentioned mediators may have a role in the sensitization of the trigeminal system and manifestation of headache secondary to SARS-CoV-2 infection. Another case-control study compared the cytokine and interleukin profile in COVID-19 patients with headache and headache-free ones (33). IL-10, IL-23, and PIGF1 levels were significantly higher in patients with headache than in COVID-19 patients without headaches. They interpreted the higher levels of the anti-inflammatory cytokine IL10 as a response to a proinflammatory cytokine storm, highlighting a more efficient immune response in this group.

### Cough Headache (4.1)

Cough headache is a rare subtype of headache that occurs following coughing, other straining maneuvers such as sneezing and bending over, or a Valsalva maneuver in the absence of any imaging abnormalities. Headache is usually bilateral, posterior, brief, and self-limiting. It typically lasts for up to 30 min but can last for up to 2 h in some individuals. In 40% of the cases, cough headache syndrome is symptomatic and occurs secondary to other disorders (16). A sudden onset headache associated with cough has also been observed in victims of COVID-19 (31, 34). In a cross-sectional study on hospitalized patients with confirmed COVID-19, cough headache was reported among 26% of those who had headache associated with COVID-19 (35). There are also reports of the frequency of 2 and 10% for cough headache patterns (34, 36). Some studies suggest that cough headache related to a systemic infection might occur as the result of vascular tone alteration in the cranial vessels (37).

### Headache Attributed to Hypoxia and/or Hypercapnia (10.1)

This headache syndrome develops and worsens after exposure to hypoxia and/or hypercapnia and improves significantly in parallel with the improvement in the O_2_-CO_2_ balance (16). In more severe cases, SARS-COV2 infection may lead to diffuse alveolar and systemic inflammation and gas exchange disorders causing hypoxia-induced neuro-inflammation and headache (38). In a cohort of hospitalized covid-19 patients, those with silent hypoxemia had new-onset headache more often (39).

### Headache Attributed to Cranial or Cervical Vascular Disorder (6)

There is growing evidence that COVID-19 is a predisposing factor for hypercoagulable state and thromboembolic events. It has been estimated that the incidence of thrombotic events is 21%−49% and the prevalence of acute cerebrovascular events is 1.5% in COVID-19 patients (40–42). However, the incidence might be higher in severe cases who require intensive care unit (ICU) admission compared to those admitted to general wards (43). Increased risk of thrombosis might be explained by endothelial damage due to cytokine release syndrome. Cerebral venous sinus thrombosis (CVST), ischemic stroke, and hemorrhagic cerebrovascular events have been reported as rare complications of COVID-19. Asif et al. (44) reported a case of a young man without any other risk factors for thrombotic events who presented with headache and extensive CVST after recovery from COVID-19 infection. Although focal neurologic signs and decreased level of consciousness are more prevalent than headache in many conditions, particularly in ischemic and hemorrhagic stroke, headache is commonly the prominent warning symptom in CVST and subarachnoid hemorrhage (ICHD-3); moreover, it is important to identify the association of these disorders with headache in patients with COVID-19 infection.

### Headache Attributed to Increased Cerebrospinal Fluid (CSF) Pressure (7.1)

According to the ICHD-3 diagnostic criteria, for a headache to be diagnosed as a headache attributed to increased CSF pressure, the CSF pressure should exceed 250 mm CSF with normal components. Moreover, at least two of the following conditions should be present: papilledema, headache developed in temporal relation to intracranial hypertension, and headache alleviated in temporal relation to reduced CSF pressure.

In a case report by Marchiori, a 35-year-old woman with a confirmed diagnosis of COVID-19 presented with a worsening headache. She became disoriented during hospitalization. Fundoscopy examination revealed papilledema. The CSF pressure was as high as 40 cmH_2_O but CSF analysis was normal (45). In a cross-sectional study of 13 patients with severe persistent headache in the course of infection who underwent CSF analysis, intracranial hypertension was seen in 84.6% (11 out of 13; considering a normal pressure of up to 200 mmH_2_O) of the patients in the absence of meningitis or encephalitis. Assuming 250 mmH_2_O as the cut-off pressure according to the revised criteria for PTCS (46), intracranial hypertension was seen in 46.1% of the patients (six out of 13) (47).

## The Possible Pathogenic Mechanisms of Headaches Secondary to SARS-CoV-2 Infection

### Direct Involvement of Trigeminal Nerve in Nasal Cavity by Virus

Angiotensin-converting enzyme II (ACE2) -a metallopeptidase- is the specific SARS-CoV-2 receptor for cell entry. Several studies have demonstrated that SARS-COVs can cause multiple organ damage through downregulation of ACE2 expression on cells. Studies investigating influenza also support this mechanism (48). In the CNS, ACE2 is mainly expressed in neurons but it is also expressed in the glia. It is particularly present in the brain stem and motor cortex, caudoputamen, thalamus, raphe nucleus, tractus solitarius, rostral ventrolateral medulla, and nucleus ambiguous (49–52). Some studies hypothesized that the presence of the renin-angiotensin system (RAS) in the trigeminal ganglia could support the direct invasion of trigeminal nerve ending through the nasal or oral cavity (52, 53). *In vivo*, SARS-CoV studies have revealed that the virus invades the central nervous system through the olfactory bulb and then spreads retrogradely to other areas *via* the trans-synaptic route. ACE-related and olfactory routes can also be candidate pathways for neuroinvasion of the virus (54).

### Endothelial Cells and ACE2 Receptor

High expression of ACE2 in the capillary endothelium is suggestive of vascular attack and trigeminovascular activation leading to headache (38). SARS-CoV-2 spike protein interacts with ACE2 on the capillary endothelium and impairs the blood-brain barrier (55). Activation of the trigeminovascular system leads to the release of pain-generating neurotransmitters such as substance P and calcitonin gene-related peptide (CGRP), which might generate headaches that are similar in presentation to migraine with throbbing pain, nausea, photophobia, and phonophobia (56, 57).

### Pro-inflammatory Mediators and Cytokines

Another theory underlying the pathogenesis of COVID-19-associated headache includes a consequence of circulating pro-inflammatory mediators (52). Cytokines are involved in the modulation of the pain threshold and sensitization of the trigeminal nerve fibers (58–61). Headache is usually accompanied by other symptoms of systemic infection in the course of the disease, which is suggested to be provoked by cytokine release syndrome (CRS) which occurs because of an aggressive immune response to infection. Cytokine release syndrome is characterized by a cytokine storm with an increase in interleukins (IL)-2, IL-6, IL-7, and IL-10, tumor necrosis factor-alpha (TNFα), interferon-γ inducible protein 10, monocyte chemoattractant protein 1, granulocyte-colony stimulating factor, and macrophage inflammatory protein 1-α (62–65).

Previous studies have described the role of cytokines such as TNFα in headache pathophysiology (66). Evidence supports the role of TNFα in headache pathogenesis, including increased TNFα serum level during a migraine attack (58) and headache induction following TNFα infusion (67). Some studies have reported an increase in the levels of inflammatory cytokines including IL-1, which has many activities in common with TNFα. INF- γ may also cause headaches to a lesser extent (68, 69). In another study, patients treated with GM-CSF experienced headaches (70). Furthermore, headache has been recognized as a dominant side effect of IL-2 (71). Additionally, experimental models have demonstrated the secretion of pro-inflammatory mediators following the infection of macrophages and glial cells by coronaviruses. However, it is unconvincing to explain early and the isolated manifestation of headache through cytokine storm, and further data are needed (17).

## Post Vaccination Headache

Headache is among the most frequent adverse effects following coronavirus disease 2019 (COVID-19) immunization. It is reported by approximately half of the vaccine recipients, both in clinical trials and in real-world data (72). Headache typically presents within the first 72 h post-vaccination and may be associated with additional symptoms, such as fatigue, fever, myalgia, arthralgia, or diarrhea (72). Although any vaccine is expected to cause temporary side effects, the results of a meta-analysis investigating the safety of different types of vaccines for COVID-19 indicated that mRNA-based vaccines had higher rates of side effects including headache compared to adenovirus-based, inactivated, and pro-subunit vaccines (73). In a study, the cumulative rate of headache/migraine episodes after receiving all COVID-19 vaccines was reported as 2.25-fold higher than the daily frequency of headache disorders (15). The exact phenotype of vaccination-associated headaches especially its duration is unclear so far. The majority of evidence indicated that vaccine causes temporary attacks that lasted <24 h (74). However, there is some evidence that reported persistent headaches attributed to COVID-19 vaccination for up to 21 days (13, 75).

The headache could be the most frequent symptom of cerebral venous sinus thrombosis (CVT), and it may occur as the exclusive symptom or accompanied by other manifestations (76–78).

So, in patients with a new-onset constant headache, about 1 week after immunization, evaluation for CVT is highly recommended (74). In the case of thrombosis accompanied by thrombocytopenia syndrome which was recently discussed, treatment by intravenous immunoglobulins might be the preferred treatment to overcome the immune basis of the phenomenon, and treatment with anticoagulant and platelet infusion should be avoided (79).

## Other Headache Types That are not due to COVID-19 Infection But are More Frequently Seen in the COVID-19 ERA (According to ICHD-3 Classification)

### Headache Attributed to Refractive Error

In patients with uncorrected refractive error of one or both eyes, the headache might be aggravated by prolonged visual tasks. Since people spend more time on electronic devices during isolation in the COVID-19 era, this type of headache may occur in individuals with pre-existing refractory errors (16).

### External-Compression Headache as Headache Associated With Personal Protective Equipment (PPE)

Health care providers working in high-risk areas are at a greater risk of developing headaches while wearing face masks or protective eyewear or both resulting from sustained compression of pericranial soft tissue. Such headaches are mostly reported to be mild, bilateral, and variable in location (80). Ong et al. studied 158 frontline workers who wore N95 face masks with or without eye protection in Singapore and found that 81.0% of them (*n* = 128) described de-novo PPE-associated headaches. The location was bilateral in all of the participants and corresponded to the contact areas of the protection equipment. In the majority of the participants, headache developed and was relieved within 60 min of wearing N95 face masks (75.0%) and protective eyewear (82.2%), thus fulfilling the diagnostic criteria for external compression headache (81). Another cross-sectional study by Jafari et al. on 243 frontline healthcare workers showed that 72.4% of them reported headaches after the use of masks, with the N95 mask being the most commonly reported cause of headaches (82). Accordingly, a systematic review and meta-analysis of 14 studies investigated the effect of PPE use on the physical health of 11,746 health care workers during the COVID-19 (83). On the other hand, the results of a national web-based survey on the general population indicated that 97% of participants with pre-existing headaches had worsening attacks and 56% of the study population developed *de novo* headaches following PPE use (84).

### Headache Attributed to Neck Disorders

Involvement of any structure in the neck including the soft tissue and muscular or bony structures can contribute to headache, which is usually accompanied by neck pain (16, 85). Neck pain is specifically known as a possible trigger or part of the primary headaches including migraine (86). It has been shown that neck pain has worsened during the lockdown, which can be attributed to poor posture and increased screen time on electronic devices (87). In a study conducted on the home working population during the COVID pandemic, 23.5% reported neck pain (88). Due to the potential increase in neck disorders during the COVID-19 pandemic and subsequent headaches, it is essential to consider this type of headache. Haghdoost et al. (89) investigated the impact of the COVID-19 pandemic among over 100,000 users of migraine-specific phone applications and reported that neck pain served as the four commonest headache triggers in 2018, 2019, and 2020.

## Primary Headaches Course During COVID-19 Infection

According to existing evidence, headache characteristics in the symptomatic COVID-19 patients are sudden to gradual onset, resistance to common analgesics, or high relapse rate, and limited to the active phase of the COVID-19 (52). The results of a cross-sectional study on 3,458 participants indicated that COVID-19-positive patients had a bilateral headache, lasting over 72 h, and a low response to analgesics compared to those without COVID-19 (90). Another recent study aimed to determine specific characteristics of COVID-19-associated headache among 287 moderately affected hospitalized patients, prospectively. Using the Latent class cluster analysis 2 distinct class of headache phenotypes were identified in COVID-19 patients. In cluster 1, patients were characterized by higher intensity, frequency, duration of attacks, unresponsive to paracetamol, pulmonary involvement, and IL-6 level compared to Cluster 2. According to the ROC curve cutoff values they suggested that VAS >70 severity, >9 h duration, >5 headache days, and IL-6 >43 pg/ml levels can be diagnostic for COVID-19 headache (91). Interestingly, besides the migraine-like characteristics, headaches associated with COVID-19 could be accompanied by unusual sensory symptoms including anosmia and gastrointestinal symptoms such as diarrhea (17, 90).

## Expert Opinion on the Treatment of Headache in COVID-19

According to our experience with COVID-19 patients, headache in the early phase of COVID-19 infection usually occurs in association with mild systemic symptoms and responds well to proper hydration, non-steroid anti-inflammatory drugs, or high dose acetaminophen. However, treatment is better to be continued for at least a few days to prevent headache recurrence. Headaches after vaccination and omicron variant of the virus are also common, but usually remit after a few days and the suggested treatments are the same, but it is necessary to pay more attention to headaches start 1 week after vaccination and should be evaluated for CVT if clinically indicated. In many patients, anxiety and concerns about the disease may also provoke or worsen the headache. Thus, it might be favorable to add an anxiolytic drug to analgesic mediation. The headache in the second week of the disease is usually accompanied by more severe systemic symptoms and hyperimmune response. In this scenario, headache is often severe and may not respond to simple or combined analgesics. The headache in the later phase of the disease may fulfill the criteria for intracranial hypertension and corticosteroids might be helpful for a short period if there is no contraindication. Headache may also be due to the side effects of drugs used to treat COVID-19 patients such as hydroxychloroquine (92, 93), baricitinib (94, 95), and corticosteroids (96).

Apart from headaches due to COVID-19 infection, there is also an increase in the number of non-affected headache patients in clinics with or without a previous history of migraine or tension-type headache. The most prevalent headache complaints seem to be due to cervical muscle spasms secondary to the prolonged fixed position of the neck while using electronic devices as these devices are more frequently used in our new lifestyle in the COVID-19 era. Contraction of cervical muscles may cause a new headache or aggravate previous tension or migraine headaches. Stress and anxiety of isolation or fear of COVID-19 infection, as well as emotional and economic problems secondary to losing jobs or lockdown, may be other common important causes for worsening of previous headaches. Thus, public awareness of the causes of headaches, encouraging people to manage stress and maintain the correct position when using electronic devices, regular sleep, a healthy diet, and regular physical activity will help with headache prevention. Access to physicians and utilization of medical and psychological counseling, especially using telecommunication technology, is very important in controlling headaches and the preventing of chronicity.

Another issue requiring attention is a headache in medical staff, which seems to occur mainly as a consequence of increased work hours, especially in difficult stressful situations requiring wearing personal protection equipment. The medical staff should receive more attention from senior officials to reduce other types of pressures as much as possible.

## Conclusion

Headache has been identified as a common neurologic manifestation of COVID-19 infection. The exact pathogenic mechanism of headache in COVID-19 infection is not clear. Several mechanisms have been proposed to explain trigeminovascular activation and headache during infection, including direct invasion of the virus, cytokine production, ACE2 pathway, and hypoxia. When facing a headache, it is very important to use the ICHD-3 criteria to make an accurate diagnosis, which also helps to better understand the pathophysiology of the headache and explore possible treatment options. However, many aspects of headaches related to COVID infection remain poorly understood and further studies need to be carried out to improve our understanding of the headache mechanism, headache type, and the best treatment options.

## Author Contributions

Study conception and design: MT and NY. Draft manuscript preparation: NY, SH, and ZS. The final version of manuscript: MT and FM. All authors contributed to the article and approved the submitted version.

## Conflict of Interest

The authors declare that the research was conducted in the absence of any commercial or financial relationships that could be construed as a potential conflict of interest.

## Publisher's Note

All claims expressed in this article are solely those of the authors and do not necessarily represent those of their affiliated organizations, or those of the publisher, the editors and the reviewers. Any product that may be evaluated in this article, or claim that may be made by its manufacturer, is not guaranteed or endorsed by the publisher.
